# Outcomes of Laparoscopic Surgery in Very Elderly Patients with Colorectal Cancer: A Survival Analysis and Comparative Study

**DOI:** 10.3390/jcm12227122

**Published:** 2023-11-15

**Authors:** Nicola Passuello, Lino Polese, Giulia Ometto, Ugo Grossi, Enzo Mammano, Fabrizio Vittadello, Alvise Frasson, Emanuela Tessari, Patrizia Bartolotta, Dario Gregori, Giacomo Sarzo

**Affiliations:** 1 OSA General Surgery, Padua University Hospital, 35128 Padua, Italy; nicola.passuello@aopd.veneto.it (N.P.); giulia.ometto@studenti.unipd.it (G.O.); enzo.mammano@aopd.veneto.it (E.M.); fabrizio.vittadello@aopd.veneto.it (F.V.); alvise.frasson@aopd.veneto.it (A.F.); emanuela.tessari@aopd.veneto.it (E.T.); giacomo.sarzo@aopd.veneto.it (G.S.); 2 Department of Surgery, Oncology and Gastroenterology, University of Padua, 35128 Padua, Italy; lino.polese@aopd.veneto.it; 3 Surgery Unit 2, Regional Hospital Treviso, 31100 Treviso, Italy; 4 Unit of Biostatistics, Epidemiology and Public Health, Department of Cardiac Thoracic Vascular Sciences and Public Health, University of Padua, 35121 Padua, Italy; patrizia.bartolotta@ubep.unipd.it (P.B.); dario.gregori@ubep.unipd.it (D.G.)

**Keywords:** laparoscopic surgery, colorectal cancer, elderly patients, survival analysis, multidisciplinary care

## Abstract

(1) Background: Colorectal cancer (CRC) is a global health concern, particularly among the elderly population. This study aimed to assess the impact of laparoscopic surgery on CRC patients aged ≥80 years. (2) Methods: We conducted a retrospective analysis of prospectively collected data from consecutive CRC patients who underwent surgery at our institution between July 2018 and July 2023. The patients were categorized into three groups: those aged over 80 who underwent laparoscopic surgery (Group A), those aged over 80 who underwent open surgery (Group B), and those under 80 who underwent laparoscopic surgery (Group C). We examined various clinical and surgical parameters, including demographic data, medical history, surgical outcomes, and survival. (3) Results: Group A (N = 113) had shorter hospital stays than Group B (N = 23; *p* = 0.042), with no significant differences in complications or 30-day outcomes. Compared to Group C (N = 269), Group A had higher comorbidity indices (*p* < 0.001), more emergency admissions, anemia, low hemoglobin levels, colonic obstruction (*p* < 0.001), longer hospital stays (*p* < 0.001), and more medical complications (*p* = 0.003). Laparotomic conversion was associated with obstructive neoplasms (*p* < 0.001), and medical complications with ASA scores (*p* < 0.001). Both the medical and surgical complications predicted adverse 30-day outcomes (*p* = 0.007 and *p* < 0.001). Survival analysis revealed superior overall survival (OS) in Group A vs. Group B (*p* < 0.0001) and inferior OS vs. Group C (*p* < 0.0001). After a landmark analysis, the OS for patients aged 80 or older and those under 80 appeared to be similar (HR 2.55 [0.75–8.72], *p* = 0.136). (4) Conclusions: Laparoscopic surgery in very elderly CRC patients shows comparable oncological outcomes and surgical complications to younger populations. Survival benefits are influenced by age, comorbidities, and medical complications. Further prospective multicenter studies are needed in order to validate these findings.

## 1. Introduction

Colorectal cancer (CRC) holds significant socio-sanitary importance worldwide. It is ranked as the third most common cancer in terms of both incidence and mortality, following breast and lung cancer among women and prostate and lung cancer among men [1,2].

In 2020, Italy witnessed an estimated 45,000 new cases of CRC (24,000 in men and 21,000 in women) [3]. Aging has emerged as a pivotal factor in the development of CRC, with its prevalence steadily increasing over the past decade, particularly among individuals aged 65 and older [4,5].

Approximately 90% of newly diagnosed cases occur in individuals aged 50 and above, with 60% of them being older than 65 [6]. The prognosis for CRC has improved significantly due to early detection and advancements in clinical management. Over the last two decades, there has been a notable increase in 5-year survival rates, particularly among patients with advanced tumors [7].

The first laparoscopic colectomy was introduced by Jacobs in 1991 [8]. Minimally invasive colorectal surgery offers numerous advantages, including smaller incisions, improved aesthetic outcomes, reduced postoperative pain, quicker recovery of intestinal function, shorter hospital stays, and lower postoperative mortality and morbidity rates, while also maintaining comparable oncological outcomes compared to open surgery [9,10].

The safety and feasibility of minimally invasive techniques have been substantiated by various randomized trials [11,12,13]. Nevertheless, in clinical practice, laparoscopic surgery has not been universally adopted for very elderly patients due to the prolonged operative times and the potential adverse effects of pneumoperitoneum on heart and lung function, especially in patients with a higher Charlson Comorbidity Index (CCI), such as the very elderly population (those over 80 years old).

Despite these considerations, there is evidence demonstrating the safety of laparoscopic techniques in oncologic surgery for the elderly population (aged over 75) [14,15,16,17,18,19]. However, the role of minimally invasive surgery for CRC in very elderly patients (aged over 80) with comorbidities remains undefined.

The aim of this study was to assess the impact of the laparoscopic surgical approach on patients aged ≥80 years.

## 2. Materials and Methods

A retrospective analysis of prospectively collected data was conducted in accordance with the STrengthening the Reporting of OBservational studies in Epidemiology (STROBE) guidelines.

All patients who underwent colonic or rectal resection for CRC at the OSA General Surgery of Padua University Hospital between July 2018 and July 2023 were eligible for inclusion.

The inclusion criteria encompassed patients aged ≥80 years who underwent either laparoscopic (Group A) or laparotomic (Group B) surgery for CRC, as well as patients under 80 years of age who underwent laparoscopic surgery for CRC (Group C). Patients under 80 who underwent laparotomic surgery, transanal procedures, or surgeries unrelated to colorectal resection for CRC (e.g., liver resection for metastasis) were excluded from the study.

All patients had been recommended for oncologic radical surgery, which included various procedures such as right hemicolectomy, extended right hemicolectomy, transverse colon resection, left flexure resection, left hemicolectomy, sigmoid resection, anterior rectal resection with total mesorectal excision, abdomino-perineal amputation according to Miles’ technique, and subtotal colectomy with ileo-rectal anastomosis. These procedures were consistently performed by the same team of five surgeons using identical techniques. Trocar placement on the patients’ abdomens remained consistent for all procedures. Radical resections involved high ligation of vascular structures, such as the ileo-colic pedicle, middle colic pedicle, inferior mesenteric artery, and left colic artery, to ensure optimal lymph node radicality. In cases of anterior rectal resection, mobilization of the left flexure was consistently carried out. Palliative operations, such as colonic bypass or ileo/colostomy, were performed in cases where radical resection was deemed impossible due to tumor involvement in vital structures or multiple abdominal organs.

Follow-up was conducted through outpatient assessments 30 days after surgery, followed by assessments after 3, 6, 12, 18, and 24 months.

Data on adjuvant chemotherapy were obtained in collaboration with the Oncology Unit of the Venetian Oncology Institute (IOV), with chemotherapy protocols administered in accordance with current oncological guidelines.

In the initial phase, we compared the surgical outcomes between patients aged over 80 who underwent laparoscopy and those who underwent laparotomy. In the subsequent phase, we analyzed the surgical outcomes of patients aged over and under 80 years who underwent laparoscopy.

We collected and analyzed a range of prospectively collected data, including:

- Demographic information;
- Medical history and Charlson Comorbidity Index (CCI);
- Clinical symptoms at presentation;
- Pre-operative findings (e.g., colonoscopy, computed tomography, pelvic magnetic resonance imaging for rectal tumors);
- Perioperative data (type of resection, laparoscopy/open);
- Classification as an elective or emergent operation;
- Incidences of conversion to laparotomy;
- Post-operative medical and surgical complications, including those classified as Clavien–Dindo grade >2;
- Length of hospital stay;
- 30-day outcome, categorized as deceased, discharged, or still hospitalized;
- Sample histology and immunohistochemistry;
- Disease stage;
- Any adjuvant treatment;
- Signs and timing of cancer recurrence during follow-up;
- Causes and timings of patient deaths.

In a subsequent phase of the study, we aimed to identify potential risk factors for adverse outcomes in the laparoscopic groups (group A and group C), including laparotomic conversion, the onset of severe medical complications, death, and hospitalization exceeding 30 days. Data collection was conducted using the Research Electronic Data Capture (REDCap) platform [20], and the data were analyzed using Jamovi version 2.3 [21].

### Statistical Analysis

Statistical comparisons involved the χ^2^ test or Fisher’s exact test for categorical variables, as well as the Wilcoxon–Kruskal–Wallis test and the signed-rank Wilcoxon test for continuous variables.

We employed univariable and multivariable logistic regression tests to identify potential risk factors for laparotomic conversion, medical and surgical complications, and adverse 30-day outcomes. Results were presented as odds ratios (ORs) with 95% confidence intervals (CI). A *p*-value of <0.05 was considered statistically significant for all analyses. Statistical analysis was performed using the R system, version 4.1.0, and Gt summary.

Follow-up data were evaluated for overall survival (OS) and disease-free survival (DFS) using Kaplan–Meier curves, with comparisons made at 12, 24, and 36 months through landmark analysis. Hazard ratios (HR) with 95% confidence intervals (95% CI) were calculated, and Cox proportional hazard models were constructed to assess the impact of various factors on survival outcomes.

## 3. Results

### 3.1. Patient Group Selection and Demographics

In a comprehensive database of 435 patients who underwent CRC surgery, we excluded 15 cases involving transanal procedures and 15 open surgeries in patients under the age of 80. The remaining patients were categorized into three groups: patients aged over 80 who underwent laparoscopic surgery (N = 113, group A), patients aged over 80 who underwent open surgery (N = 23, group B), and patients under 80 who underwent laparoscopic surgery (N = 269, group C).

### 3.2. Comparison of Group A and Group B

Table 1 illustrates the differences between group A and group B. In this comparison, we noted shorter hospital stays among the patients in group A (10 (7–15) days vs. 14 (11–18) days, *p* = 0.04).

We did not identify any significant differences in the initial symptoms of tumor onset, the occurrence of severe postoperative medical or surgical complications (Clavien–Dindo > 2), or the rate of anastomotic leakage. Moreover, there was no significant variation in the 30-day outcomes. Additionally, we observed comparable lymph node radicality (19 (15–26) in group A vs. 14 (11–22) in group B, *p* = n.s.). Complication analysis did not reveal significant differences between group A and group B (Table 2).

### 3.3. Comparison of Group A and Group C

In the next step, we analyzed the distinctions between patients in group A and those in group C, as displayed in Table 3. Patients in group A were more frequently admitted from the Emergency Department or other hospital wards and had a higher CCI (*p* < 0.001). They also presented more often with malignancies associated with anemia, low hemoglobin levels (*p* < 0.001), or colonic obstruction (*p* < 0.001). The conversion rate was 17/113 (15%) cases in group A compared to 14/269 (5.2%) in group C (*p* = 0.003). Patients in group A experienced longer hospital stays (10 (7–15) days vs. 8 (6–10) days, *p* < 0.001). In the postoperative period, we observed a higher incidence of severe medical complications in group A (*p* = 0.003), while the rates of surgical complications and anastomotic leakage were similar between the two groups (*p* = n.s.). A higher incidence of 30-day mortality was also noted in group C (8/113 vs. 1/269, *p* < 0.001). While there were no differences in terms of surgical complications, a significantly higher number of medical complications was observed in group A (20 [18%] vs. 18 [6.7%] in group C; *p* = 0.003).

### 3.4. Risk Factor Analysis for Laparoscopic Groups (Group A and Group C)

Table 4 presents the results of univariable and multivariable logistic regression tests using potential risk factors that showed significant associations in the univariable analysis.

**Figure 1:**
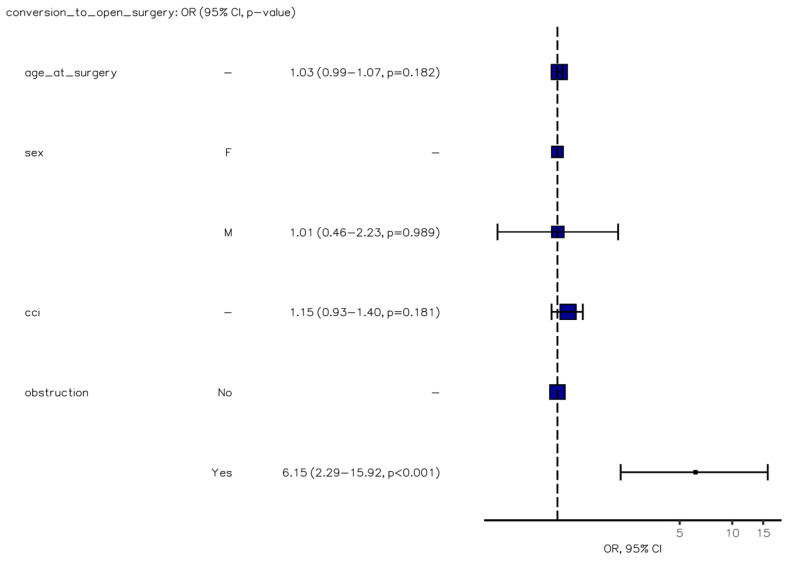
Binary logistic regression model of factors predicting conversion to open surgery.

**Figure 2:**
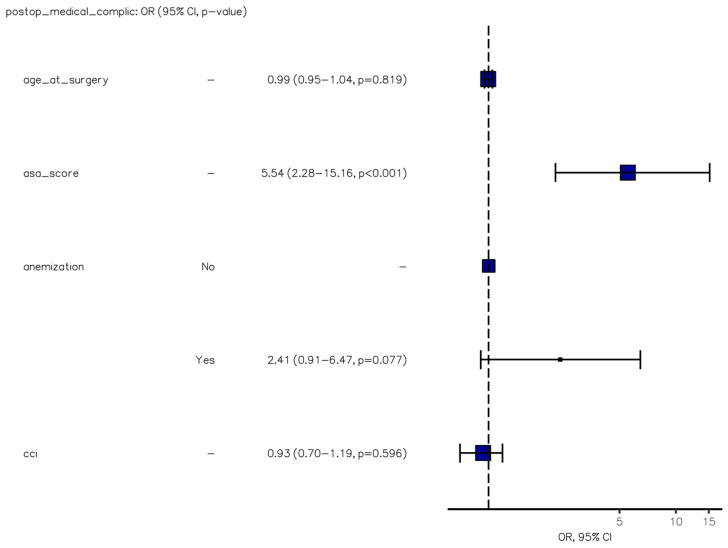
Binary logistic regression model of factors predicting postoperative medical morbidity.

**Figure 3:**
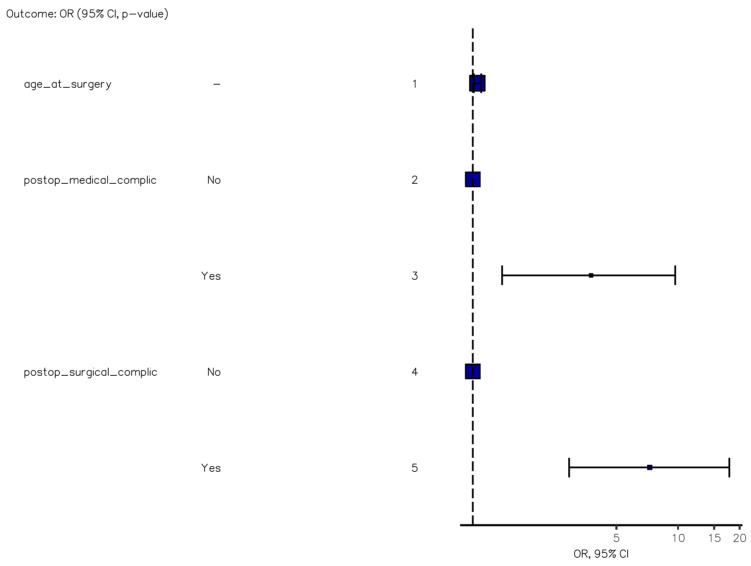
Multinomial regression model of factors predicting death or extended hospitalization at 30 days.

The presence of obstructive neoplasms emerged as an independent risk factor for laparotomic conversion (OR, 6.15 [95%CI, 2.29–15.92], *p* < 0.001) (Figure 1), while medical complications were strongly correlated with the American Society of Anesthesiology (ASA) score (OR, 5.54 [2.28–15.16], *p* < 0.001) (Figure 2). Furthermore, both medical and, particularly, surgical complications were identified as negative independent prognostic factors for death or extended hospitalization at 30 days (*p* = 0.007 and *p* < 0.001) (Figure 3). Age at surgery exhibited a significant association with all three adverse outcomes mentioned above in the univariable analysis. No potential risk factors for major surgical complications (Clavien–Dindo grade >2) were identified in the logistic regression analysis.

### 3.5. Survival Analysis

**Figure 4:**
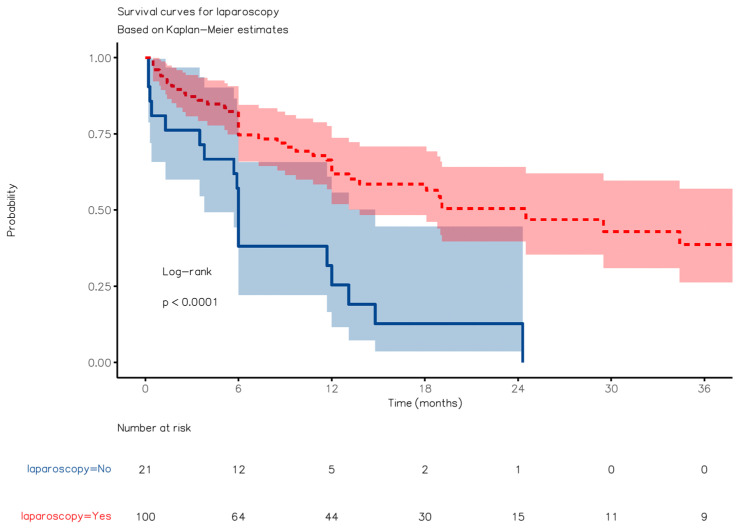
Kaplan–Meier overall survival curve with comparisons between Groups A and B.

**Figure 5:**
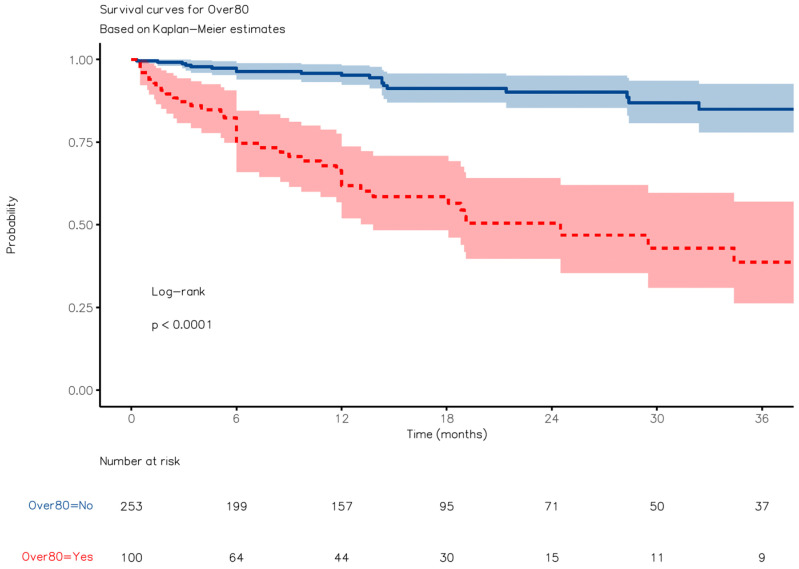
Kaplan–Meier overall survival curve with comparisons between Groups A and C.

**Figure 6:**
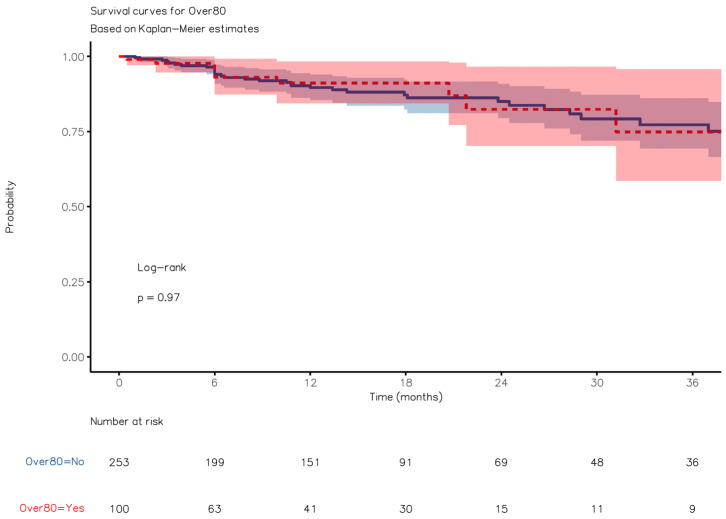
Kaplan–Meier disease-free survival curve with comparisons between Groups A and C.

The median follow-up lasted 10 (3–19) months for group A, 6 (4–12) months for group B, and 13 (6–26) months for group C. The patients in group A exhibited superior OS compared to those in group B (24.2 months [95%CI: 14.2–33.01] vs. 6.1 months [4.2–14.8], respectively; HR, 0.31 [95%CI, 0.18–0.55], *p* < 0.0001) over a median follow-up period of 12 (6–24) months (Figure 4). However, they displayed worse OS than those in group C (HR, 6.21 [3.69, 10.44]; *p* < 0.0001) (Figure 5). The observed survival difference between group A and group C was primarily evident in the left portion of the survival curve. After a landmark analysis, the OS for patients aged 80 or older and those under 80 appeared to be similar (HR 2.55 [0.75–8.72], *p* = 0.136). Disease-free survival (DFS) showed no significant difference between group A and group C (Figure 6, *p* = n.s.).

## 4. Discussion

Elderly patients often exhibit diminished physiological functions, elevated ASA scores, and multiple comorbidities, placing them at a higher risk of post-operative morbidity and mortality [22]. Age itself is an independent risk factor for both in-hospital morbidity and post-hospital mortality in surgical patients [23]. Additionally, elderly patients are more likely to present with advanced disease or acute conditions necessitating urgent surgery upon diagnosis [24]. In such cases, a multidisciplinary approach becomes essential in order to optimize the management of pre-existing comorbidities in preparation for major surgical interventions. Complete Geriatric Assessment (CGA) has proven valuable in identifying and assessing systemic conditions. A personalized program tailored to each patient’s situation and preferences may encompass nutritional, psychotherapeutic, pharmacological, or physical–rehabilitative interventions.

However, comprehensive geriatric evaluations that consider all clinical-prognostic components remain infrequent in the preoperative assessment of elderly CRC patients. The scarcity of randomized controlled trials assessing the benefits of preoperative geriatric assessments or multi-component interventions, coupled with methodological variability in previously published studies, contributes to the uncertainty surrounding the advantages of these programs. CGA can serve as an initial step toward establishing a multidisciplinary network that offers patients access to personalized treatment plans comprising integrated interventions [25]. Nevertheless, implementation can be challenging, particularly when very elderly patients often present with advanced and complicated tumors necessitating urgent intervention.

Other experiences have suggested that laparoscopy may lead to lower complication rates in elderly individuals [22,26], although our study did not replicate these findings, possibly due to the smaller size of the open surgery group in our experience. A multicentric Japanese study from 2015 demonstrated comparable OS and DFS in very elderly patients undergoing open or laparoscopic surgery, with both techniques achieving similar lymph node radicality [27].

Other multicentric studies have indicated that laparoscopic surgery can independently predict lower postoperative mortality in CRC patients [25], even when considering patients aged 65 or older as “elderly”. In a matched case–control study, Miguchi et al. [28] demonstrated that laparoscopic CRC surgery for octogenarians produces similar oncological outcomes to open surgery and should be considered a treatment option. A comprehensive systematic review and meta-analysis published in 2022 [29] concluded that laparoscopic surgery appears to offer more benefits than open surgery in elderly subjects with CRC and should be prioritized, provided that the required technical skills and facilities are available. This aligns with our perspective, as we believe that despite the complexity of CRC treatment and the need for a multidisciplinary approach, especially for frail and elderly patients, the routine use of laparoscopy in high-volume centers should be encouraged. Despite being significantly shorter, the relatively extended hospital stay observed in the laparoscopic group can likely be attributed to the advanced age of the patients, who tended to be frail and often had associated comorbidities.

Contrary to our study, other research [30] has found that age alone is not a significant predictor of postoperative complications, while factors such as sex, tumor location, operation time, and conversion to open surgery play more significant roles. In their propensity score–matched analysis, the authors concluded that laparoscopic surgery for CRC patients aged over 80 years is both technically and oncologically safe. We endorse the view that minimally invasive techniques not only are safe and feasible in very elderly patients, but should be recommended when performed by surgeons with adequate expertise. Our own experience suggests a potential “survival benefit” associated with laparoscopy in very elderly CRC patients, although this may have been influenced by the retrospective design of the study, resulting in similar OS between patients over and under 80 years old after the initial 12–18 months following surgery.

It is noteworthy that the majority of studies focusing on octogenarians and laparoscopic surgery for colorectal cancer have primarily involved Eastern populations [22,26]. In contrast, our study represents one of the largest datasets from Italy, offering a valuable addition to the existing literature. While we acknowledge that we did not conduct a propensity matching analysis, it is important to highlight that the patients in Groups A and B exhibited substantial overlap in nearly all clinical and demographic characteristics, with the exception of hospital stay duration. This overlap provides a solid basis for comparing surgical and oncological outcomes, allowing for robust conclusions to be drawn.

However, our study has several limitations. First, it is retrospective in nature, which introduces the possibility of selection bias. Additionally, the study was conducted at a single center, which may limit the generalizability of our findings. Furthermore, the sample size in some subgroups, particularly in group B (open surgery in patients aged over 80), was relatively small, which may have influenced the statistical power of our analyses. These limitations are inherent to the nature of the study and the available resources.

In addition to these limitations, it is important to note that the relatively short timeframe of data collection from July 2018 to July 2023 may have limited our ability to assess the long-term outcomes of CRC treatment, particularly in elderly patients. The findings of this study should be interpreted within the context of the duration of data collection. A longer follow-up period might provide a more comprehensive understanding of survival rates and complications, especially for elderly patients. To mitigate this limitation, we recommend that future research should involve extended data collection and follow-up to better evaluate the long-term outcomes of CRC treatment in the elderly population, considering their unique characteristics and challenges. Prospective, multicenter studies with larger sample sizes will be needed in the future to further explore the potential benefits of laparoscopic surgery in very elderly CRC patients and to validate our findings.

Furthermore, while we attempted to control for potential confounding variables, there may still be unmeasured factors that could impact the outcomes which we examined.

The importance of considering patients with heart failure and the potentially longer duration of surgery when operating on very elderly patients should also be highlighted. While these specific factors were not explored in our study, they are important considerations that may influence surgical outcomes in this population. Future research should investigate these aspects more comprehensively to provide a deeper understanding of the challenges and considerations when operating on very elderly patients.

## 5. Conclusions

In our experience, laparoscopic surgery for very elderly CRC patients yields comparable oncological outcomes to those of open surgery and those observed in younger individuals, along with a similar rate of surgical complications as that seen in younger populations. Despite the potential selection bias, it is essential to highlight the “survival benefit” noted in the laparoscopic group compared to the open group among very elderly patients. Worse outcomes, both short- and long-term, in very elderly CRC patients appear to be influenced by age at surgery, ASA score, CCI, and particularly the occurrence of severe medical complications, which can impact the prognosis up to 12–18 months post-surgery.

Consequently, we advocate for enhanced recovery after surgery protocols, CGA, and multidisciplinary teams for these patients whenever possible. We believe that the extensive use of laparoscopic surgery in high-volume centers with appropriate expertise should be recommended for all patients. Additionally, we support the aggressive management of medical and surgical complications, especially in very elderly patients.

## Figures and Tables

**Table 1:** Characteristics of octogenarians (N = 136) with colorectal cancer who underwent laparoscopic (Group A) or open (Group B) surgery.

Characteristic	Group A (N = 113, 83%)	Group B (N = 23, 17%)	*p*-Value
Sex			n.s.
Female	65 (58%)	14 (61%)	
Male	48 (42%)	9 (39%)	
Age at surgery	84 (81, 87)	86 (84, 90)	n.s.
Provenance			n.s.
Accident and Emergency	26 (24%)	7 (30%)	
Home	66 (60%)	7 (30%)	
Other ward/hospital	18 (16%)	8 (35%)	
Pharmacological treatment			
Antiplatelets	31 (28%)	4 (17%)	n.s.
Anticoagulants	21 (19%)	10 (43%)	n.s.
Symptoms			
Anemia	53 (47%)	9 (39%)	n.s.
Rectorrhagia	28 (25%)	6 (26%)	n.s.
Obstruction	19 (17%)	9 (39%)	n.s.
Weight loss	16 (14%)	1 (4.3%)	n.s.
Change in bowel habit	27 (24%)	6 (26%)	n.s.
Abdominal pain	31 (28%)	3 (13%)	n.s.
Hemoglobin (g/L)	107 (94, 119)	104 (92, 114)	n.s.
Type of operation			n.s.
Anterior rectal resection + TME	11 (9.7%)	0 (0%)	
Miles	5 (4.4%)	0 (0%)	
Left flexure resection	3 (2.7%)	1 (4.3%)	
Left hemicolectomy	22 (19%)	2 (8.7%)	
Other (palliation)	5 (4.4%)	1 (4.3%)	
Right hemicolectomy	42 (37%)	7 (30%)	
Extended right hemicolectomy	11 (9.7%)	3 (13%)	
Sigmoid resection (partial TME)	10 (8.8%)	5 (22%)	
Subtotal colectomy	0 (0%)	2 (8.7%)	
Transverse colon resection	4 (3.5%)	2 (8.7%)	
Length of stay (days)	10 (7, 15)	14 (11, 18)	0.04
Complications (Clavien–Dindo > 2)			
Medical	20 (18%)	6 (26%)	n.s.
Surgical	17 (15%)	1 (4.3%)	n.s.
Anastomotic leak	9 (8.0%)	0 (0%)	n.s.
30-day outcome			n.s.
Dead	8 (7.1%)	5 (22%)	
Discharged	98 (88%)	16 (70%)	
Still hospitalized	6 (5.4%)	2 (8.7%)	
pT stage (N = 127)			n.s.
T1	10 (9.3%)	0 (0%)	
T2	9 (8.4%)	3 (15%)	
T3	71 (66%)	9 (45%)	
T4a	13 (12%)	4 (20%)	
T4b	4 (3.7%)	4 (20%)	
Harvested lymph nodes	19 (15, 26)	14 (11, 22)	n.s.
Microsatellite instability (N = 127)	28 (25%)	5 (22%)	n.s.
Adjuvant chemotherapy (N = 47)	5 (12%)	0 (0%)	n.s.

**Table 2:** Types of complications among the three groups.

Surgical Complications (Clavien–Dindo >2)	Group A (N = 113)	Group B (N = 23)
Anastomotic leak	9	0
Intraluminal bleeding	2	0
Hemorrhage	2	0
Abdominal collections	3	1
Bowel obstruction	1	0
Total	17 (15%)	1 (4.3%)
	*p =* n.s.	
**Medical Complications (Clavien–Dindo > 2)**	**Group A (N = 113)**	**Group B (N = 23)**
Ileus	6	2
Pneumonia/respiratory failure	5	1
Heart failure	7	2
AKI	2	1
Total	20 (18%)	6 (26%)
	*p=* n.s.	
**Surgical Complications (Clavien–Dindo >2)**	**Group A (N = 113)**	**Group C (N = 269)**
Anastomotic leak	9	10
Intraluminal bleeding	2	2
Hemorrhage	2	2
Abdominal collections	3	2
Bowel obstruction	1	4
Ureteral leak	-	1
Pancreatic leak	-	1
Vaginal leak	-	1
Total	17 (15%)	23 (8.6%)
	*p =* n.s.	
**Medical Complications (Clavien–Dindo >2)**	**Group A (N = 113)**	**Group C (N = 269)**
Ileus	6	4
Pneumonia/respiratory failure	5	3
Heart failure	7	5
Acute kidney injury	2	1
Non-surgical sepsis	-	3
Other (medical)	-	2
Total	20 (18%)	18 (6.7%)
	*p =* 0.003	

**Table 3:** Characteristics of patients aged 80 or older (Group A) and those under 80 (Group C) with colorectal cancer who underwent laparoscopic surgery.

Characteristic	Group A (N = 113, 30%)	Group C (N = 269, 70%)	*p*-Value
Sex			n.s.
Female	65 (58%)	116 (43%)	
Male	48 (42%)	153 (57%)	
Age at surgery	84 (81, 87)	65 (57, 71)	<0.001
Provenance			<0.001
Accidents and Emergency	26 (24%)	18 (6.8%)	
Home	66 (60%)	238 (89%)	
Other ward/hospital	18 (16%)	9 (3.4%)	
Charlson Comorbidity Index	7 (6, 8)	5 (4, 6)	<0.001
Pharmacological treatment			
Antiplatelets	31 (28%)	39 (15%)	0.003
Anticoagulants	21 (19%)	22 (8.4%)	0.004
Symptoms			
Anemia	53 (47%)	42 (16%)	<0.001
Rectorrhagia	28 (25%)	66 (25%)	n.s.
Obstruction	19 (17%)	8 (3.1%)	<0.001
Weight loss	16 (14%)	17 (6.6%)	n.s.
Change in bowel habit	27 (24%)	29 (11%)	0.001
Abdominal pain	31 (28%)	42 (16%)	n.s.
Hemoglobin (g/L)	107 (94, 119)	131 (118, 142)	<0.001
Surgical treatment			n.s.
Anterior rectal resection + TME	11 (9.7%)	46 (17%)	
Miles	5 (4.4%)	15 (5.6%)	
Left flexure resection	3 (2.7%)	5 (1.9%)	
Left hemicolectomy	22 (19%)	73 (27%)	
Other (palliation)	5 (4.4%)	2 (0.7%)	
Tight hemicolectomy	42 (37%)	79 (29%)	
Extended right hemicolectomy	11 (9.7%)	22 (8.2%)	
Sigmoid resection (partial TME)	10 (8.8%)	17 (6.3%)	
Subtotal colectomy	0 (0%)	5 (1.9%)	
Transverse colon resection	4 (3.5%)	5 (1.9%)	
Conversion to open	17 (15%)	14 (5.2%)	0.003
Length of stay	10 (7, 15)	8 (6, 10)	<0.001
Complications (Clavien–Dindo > 2)			
Medical	20 (18%)	18 (6.7%)	0.003
Surgical	17 (15%)	23 (8.6%)	n.s.
Anastomotic leak	9 (8.0%)	10 (3.7%)	n.s.
30-day outcome			0.001
Dead	8 (7.1%)	1 (0.4%)	
Discharged	98 (88%)	257 (96%)	
Still hospitalized	6 (5.4%)	9 (3.4%)	
pT stage (N = 363)			n.s.
T0	0 (0%)	9 (3.5%)	
T1	10 (9.3%)	58 (22%)	
T2	9 (8.4%)	44 (17%)	
T3	71 (66%)	117 (46%)	
T4a	13 (12%)	24 (9.4%)	
T4b	4 (3.7%)	5 (2.0%)	
Harvested lymph nodes	19 (15, 26)	18 (13, 25)	n.s.
Microsatellite instability (N = 363)	28 (25%)	35 (13%)	0.005
Adjuvant chemotherapy (N = 187)	5 (12%)	55 (38%)	0.002

**Table 4:** Univariable and multivariable models.

Conversion to Open Surgery		No	Yes	OR (Univariable)	OR (Multivariable)	
Age at surgery	Mean (SD)	69.4 (12.6)	77.4 (13.4)	1.06 (1.02–1.10, *p* = 0.001)		
Charlson Comorbidity Index	Mean (SD)	5.5 (2.1)	6.9 (2.2)	1.31 (1.12–1.53, *p* = 0.001)		
Obstruction	No	325 (93.9)	21 (6.1)	-	*-*	
	Yes	17 (63.0)	10 (37.0)	9.10 (3.63–22.21, *p* < 0.001)	*6*.15 (2.29–15.92, *p* < 0.001)	
**Medical Complications (CD > 2)**		**No**	**Yes**	**OR (univariable)**	**OR (multivariable)**	
Age at surgery	Mean (SD)	69.4 (12.8)	75.6 (11.5)	1.04 (1.01–1.08, *p* = 0.006)		
ASA score	Mean (SD)	2.4 (0.6)	3.0 (0.6)	6.68 (3.08–16.73, *p* < 0.001)	5.54 (2.28–15.16, *p* < 0.001)	
Anemia	No	258 (93.1)	19 (6.9)	-	-	
	Yes	79 (82.3)	17 (17.7)	2.92 (1.44–5.90, *p* = 0.003)		
Charlson Comorbidity Index	Mean (SD)	5.5 (2.2)	6.3 (2.1)	1.15 (1.00–1.33, *p* = 0.054)		
**30-day outcome**		**Discharged**	**Dead**	**Still hospitalized**	**OR (univariable)**	**OR (multivariable)**
Age at surgery	Mean (SD)	69.4 (12.7)	86.0 (5.5)	73.4 (12.8)	1.06 (1.02–1.10, *p* = 0.002)	
Medical complications (CD > 2)	No	326 (94.8)	4 (1.2)	14 (4.1)	-	-
	Yes	29 (76.3)	5 (13.2)	4 (10.5)	5.62 (2.24–13.40, *p* < 0.001)	3.76 (1.39–9.68, *p* = 0.007)
Surgical complications (CD > 2)	No	327 (95.6)	4 (1.2)	11 (3.2)	-	-
	Yes	28 (70.0)	5 (12.5)	7 (17.5)	9.34 (3.94–21.95, *p* < 0.001)	7.28 (2.95–17.79, *p* < 0.001)

## Data Availability

The data that support the findings of this study are available from the corresponding author, U.G., upon reasonable request.
